# Assessing the thermal efficiency and emission reduction potential of alcohol-based fuel curing equipment in tobacco-curing

**DOI:** 10.1038/s41598-023-40015-w

**Published:** 2023-08-16

**Authors:** Ke Ren, Xinwei Ji, Yi Chen, Huilong Luo, Jiaen Su, Yonglei Jiang

**Affiliations:** 1 Yunnan Academy of Tobacco Agricultural Sciences, Kunming, 650031 China; 2 College of Agronomy and Biotechnology, Southwest University, Chongqing, 400715 China; 3 Kunming University of Science and Technology, Kunming, 650201 China

**Keywords:** Environmental sciences, Energy science and technology

## Abstract

So far, coal, petroleum, and natural gas are still the most widely used fuels, and the emissions of SO_2_, NO_X_ and particulate matter produced from their combustion have a serious influence on the air. Therefore, it is necessary to develop a clean fuel. In this study, the bulk curing barns were equipped with different fuel equipment, Barn A used traditional coal heating equipment; Barn B used biomass briquettes fuel (BBF) integrated heating equipment; Barn C equipped with alcohol-based fuel (ABF) heating equipment. The temperature of the outer surface of the heating equipment, the exhaust gas of the chimney, and the curing heat efficiency and energy consumption were analyzed. Compared with the barn BBF and barn coal, the barn ABF can meet the flue-cured tobacco curing highest temperature requirements of 68 °C, the accuracy of the target dry bulb temperature (DBT) curve during the curing of flue-cured tobacco was 93.4%. At the same time, during ABF combustion, the emissions of CO_2_ and CO were 40.82% and 0.19%, respectively. However, no emissions of NO_X_, SO_2_, and H_2_S were detected in the chimney exhaust. Compared with the barn BBF and barn coal, the thermal efficiency of barn ABF heating equipment in the barn was increased by 44.78% and 86.28%, respectively. Additionally, the coast per kilogram of dry tobacco was reduced by 19.44% and 45.28%, respectively. Therefore, compared to barn coal and barn BBF, the barn ABF can control temperature changes more accurately, and shows an obvious advantage in environmental protection and heat utilization efficiency.

## Introduction

Flue-cured tobacco (FT) is one of the most widely planted tobacco types in China. In the process of FT production, Tobacco curing (TC) is still the most energy-consuming link, accounting for more than 80% of the energy used in the production process of TC^1–3^. At the same time, coal is still the preferred curing fuel in most FT production areas, and more than 95% of bulk curing barn use coal for TC. The annual coal consumption is large, with curing of 1 kg dry tobacco consumes 1.5–2.0 kg of coal. In China, about 3–4 million tons of coal to TC are needed every year^4,5^. However, the emissions, including CO_2_, SO_2_, NO_X_ and particulate matter, are greatly discharged during coal combustion, causing serious pollution to the environment^6,7^. About 4–5 t of smoke and dust, 160–220 t of CO_2_, 3.4–5.6 t of SO_2_, and 1.6–2.8 t of NO_X_ will emit in a group of 20 large-scale curing barns during the TC season^8^. The annual TC is lasted from July to September. During the curing period, there are large amount of smoke and soot around the bulk curing barn, which make great negative effects on the growth and quality of nearby crops, and do harm to the health of humans and animals, resulting in chronic hazards, acute hazards, and invisible hazards^9^. Among them, the smoke and soot with the characteristics of long station in the atmosphere and long transportation distance can cause the haze^10^. In addition, coal is a non-renewable resource, and there are many problems during coal burning, such as insufficient combustion, high-temperature exhaust from the chimney, and slow temperature rise that leads to a decline in the quality of FT leaves. Therefore, it is of great significance to introduce a clean energy for energy conservation, environmental protection and TC.

Alcohol-based fuel (ABF), a kind of liquid fuel based on alcohols (methanol CH_3_OH, ethanol C_2_H_5_OH, butanol C_4_H_9_OH), is derived from biomass fermentation and fossil fuels, such as coal, petroleum, and natural gas. It is recognized as a new type of renewable fuel by many countries^11–14^. Due to the gradual exhaustion of petrochemical energy, ABF is the most potential new alternative energy. In the process of agricultural production, the biomass resources including corn, straw and sugar beet are abundant. With the development of the synthesis of ABF technology using non-grain biomass as raw materials (including fermentation or gasification with a posterior processing of synthesis gas), the development of biomass ABF has been significantly improved^15–18^. Therefore, ABF with the advantages of high combustion heat value, low price, clean and environmentally friendly, wide application range, safety and reliability are expected to become a new type of energy to replace fossil fuel^19^. Since ABF have a self-oxygen supplying effect during the combustion process, compared with coal, coal tar, heavy oil, diesel, gasoline and other fuels, ABF is the most thoroughly burned fuel. The combustion emissions of ABF are mainly H_2_O and CO_2_, and the exhaust gas emissions are more than 80% lower than that of liquefied petroleum gas. It is the cleanest, most environmentally friendly and most promising fuel in the future. At present, ABF is widely used in engine fuels, industrial power generation, and heating^20,21^. In particular, alcohol-based fuels can be mixed with diesel and biodiesel fuel into excellent industry and transportation mixed fuels, among which butanol can significantly improve the combustion state of the mixed fuel formed with diesel, and has good effects in improving temperature control ability and reducing CO and NOx emissions^22^. Kilic et al.^23^ showed that when low (up to 30%) butanol is formed into fuel with diesel in flame tube boilers, combustion efficiency can be improved, which has a positive prospect for reducing emissions and improving combustion efficiency.

With the increasing awareness of energy conservation, low-carbon development and environmental protection, there were many studies on the different fuels to TC and there were mainly focus on biomass fuels, heat pumps, biogas, and solar energy^24,25^. However, there are also certain some shortcomings. For example, the use of electric energy can cause excessive pressure on the grid and high promotion costs; Solar energy will be affected by weather factors and cannot provide continuous heating throughout the day, and can only be used as an auxiliary heat source; The BBF has low energy density, ash accumulation, slagging, and tar production still exist in the combustion process^4,26,27^. Therefore, clean energy ABF are gradually being used in TC research. However, the application of ABF to TC is still in the preliminary exploration stage in China. Although there was perfect ABF curing equipment in foreign countries, it cannot be adapted to local conditions due to the production status of TC in China, the structure of bulk curing barn and the specific curing process. We predict that the use of ABF can accurately control temperature and has obvious advantages in environmental protection and heat utilization efficiency, when compared with coal and BBF. Therefore, based on the curing conditions in China and the characteristics of ABF, development of a set of ABF integrated curing equipment to efficiently use ABF energy and provide sufficient heat for curing is of significance. At the same time, it can also greatly reduce environmental pollution problems, and provide theoretical basis and technical support for sustainable development for the research and application of heating equipment in tobacco areas and the drying process of other agricultural and sideline products.

## Materials and methods

### Energy exchange in the combustion of alcohol-based systems

**Figure 1:**
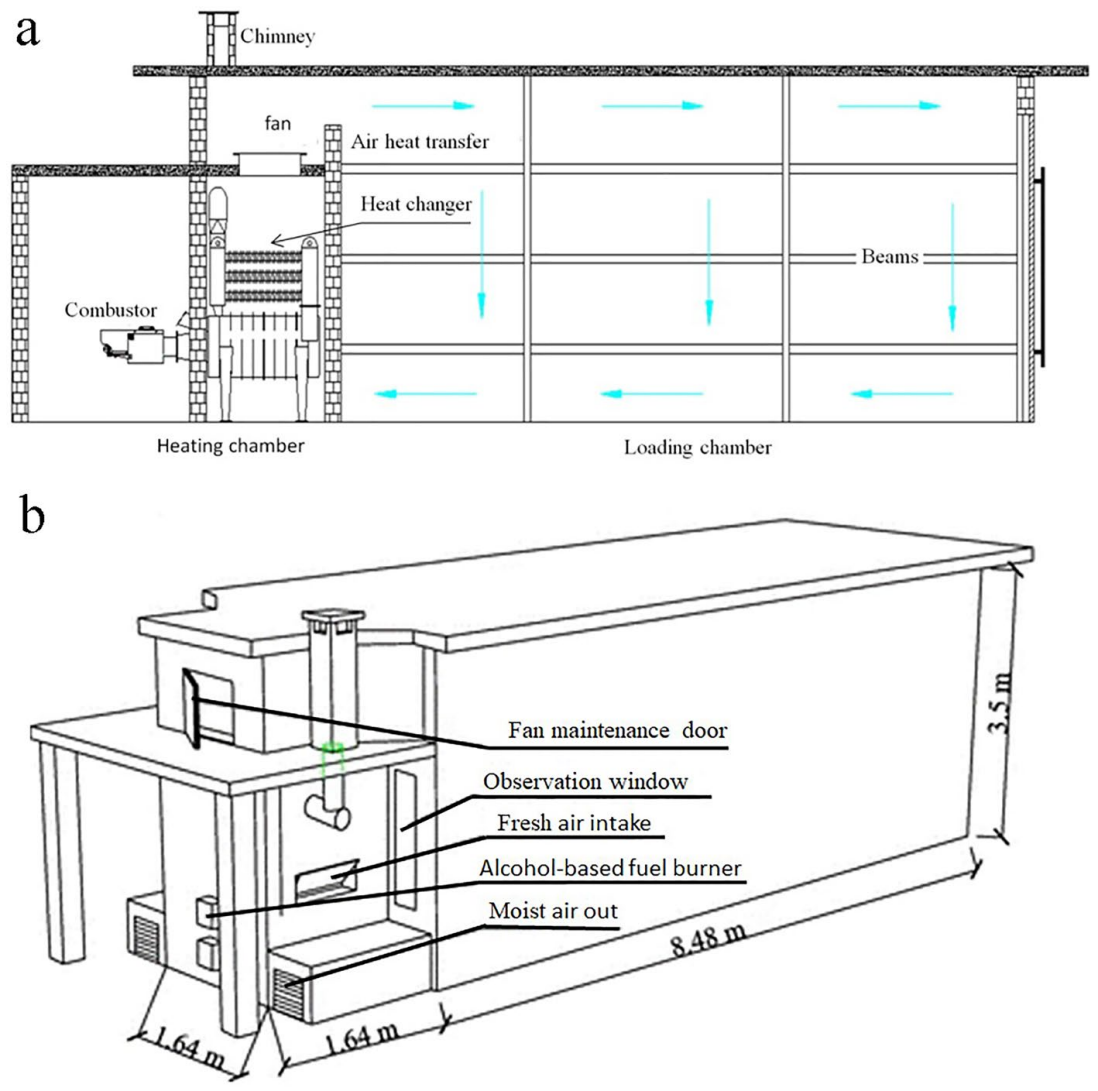
Bulk curing barn drop of alcohol-based fuel. (**a**) Gas flow diagram. (**b**) Main equipment distribution.

In 2009, the basic shape and structure of the coal heated air rising and air falling bulk curing barn were established by the China Tobacco. The ABF equipment was designed to be improved on the basis of the original coal bulk curing barn and the characteristic of ABF (Fig. 1). ABF supplied by Yunnan Xianfeng Chemical Industry Co., Ltd. The density of ABF is 791 kg/m^3^, the lower heating value is 23.36 MJ/kg, the flash point is 11 °C, the ignition temperature is 385 °C, and the alcohol content is 70%. The supply of heat involves the following formulas.

Curing barn system thermal efficiency (η) is expressed by Eq. (1), which can reflect the efficiency of fuel utilization,

1 $$ \eta \, = \,\left( {{\text{m}}_{{1}} - {\text{ m}}_{{2}} } \right)\, \times \,{\text{C}}_{{\text{w}}} \times { 1}00\% /{\text{m}}_{{3}} \times {\text{ Q}}_{{2}} , $$

where m_1_ (kg) is the amount of fresh tobacco per curing, m_2_ (kg) is the amount of dry tobacco per curing, C_W_ is the water loss energy dissipation constant of tobacco leaves during curing, the value of which is 2.6 × 10^3^ kJ/kg, m_3_ (kg) is the amount of fuel consumption, and Q2 (KJ/kg) is the calorific value of ABF combustion^28^.

Q1 (KJ/h) is the maximum energy consumption per hour per batch in per bulk curing barn is given by Eq. (2).

2 $$ {\text{Q}}_{{1}} = {\text{ m}}_{{1}} \times \, \alpha \, \times {\text{ C}}_{{\text{w}}} \times \, \beta /\eta , $$

where α (%) is the water loss rate of tobacco leaves after curing, which is obtained by the difference between fresh tobacco and dry tobacco per curing room, β (%) is the maximum water loss rate per unit time of tobacco leaves during curing, which is obtained by comparing the continuous sampling and weighing of tobacco leaves during curing. η (%) is the system thermal efficiency of the bulk curing barn.

m_4_ (kg) is the maximum amount of ABF required per hour for TC by the combustor is given by Eq. (3).

3 $$ {\text{m}}_{{4}} = {\text{ Q}}_{{1}} /\Delta h, $$

where ∆*h* (MJ/kg) is the combustion value of ABF, the value of which is 23.36 MJ/kg.

The heat released by ABF combustion in the combustor is transferred through the heat exchanger, and the high temperature air passes through convection and radiation to the outer wall of the heat exchanger, and then passes the heat to the air through the heat exchanger. Finally, the heat is transferred to dry tobacco leaves by convection. The results show that the main heat transfer modes are heat conduction and convection, and the radiation heat transfer can be neglected.

Q_h_ (KJ/h) is the heat transfer through the heat conduction of the heat exchanger, calculated using Eq. (4)

4 $$ {\text{Q}}_{{\text{h}}} = \,\lambda \, \times \,{\text{F}}\, \times \,\frac{tb1 - tb2}{{\updelta }}, $$

where δ is the wall thickness of the heat exchanger, its value is 2.75 mm, F is the heating area of the heat exchanger, and the t_b1_ and t_b2_ are the internal temperature and external temperature of the heat exchanger respectively. λ is the thermal conductivity of the heat exchanger, which is determined by the heat exchanger material and its value is 163.29 kJ/(m·h °C).

Q_c_ (KJ/h) is heat transfer from heat exchanger through convection heat transfer, calculated using Eq. (5).

5 $$ {\text{Q}}_{{\text{c}}} = \, \alpha \, \times {\text{ F }}\left( {{\text{ t}}_{{1}} - {\text{ t}}_{{2}} } \right), $$

where α is an exothermic factor with values of 20–100 kcal (m·h °C). t_1_ (°C) is the internal flue gas temperature of the heat exchanger, t_2_(°C) is the outer wall temperature of the heat exchanger.

Considering the comprehensive heat transfer of heat conduction and convection, the Q_s_ (KJ/h) is the combined heat transfer between heat conduction and convection, calculated using Eq. (6).

6 $$ {\text{Q}}_{{\text{s}}} = \,\frac{{{\text{t}}1 - {\text{t}}2}}{{\frac{1}{{{\upalpha }1}} + \frac{\delta }{\lambda } + \frac{1}{{{\upalpha }2}}}}. $$

### Design and construction of alcohol-based barn

**Figure 2:**
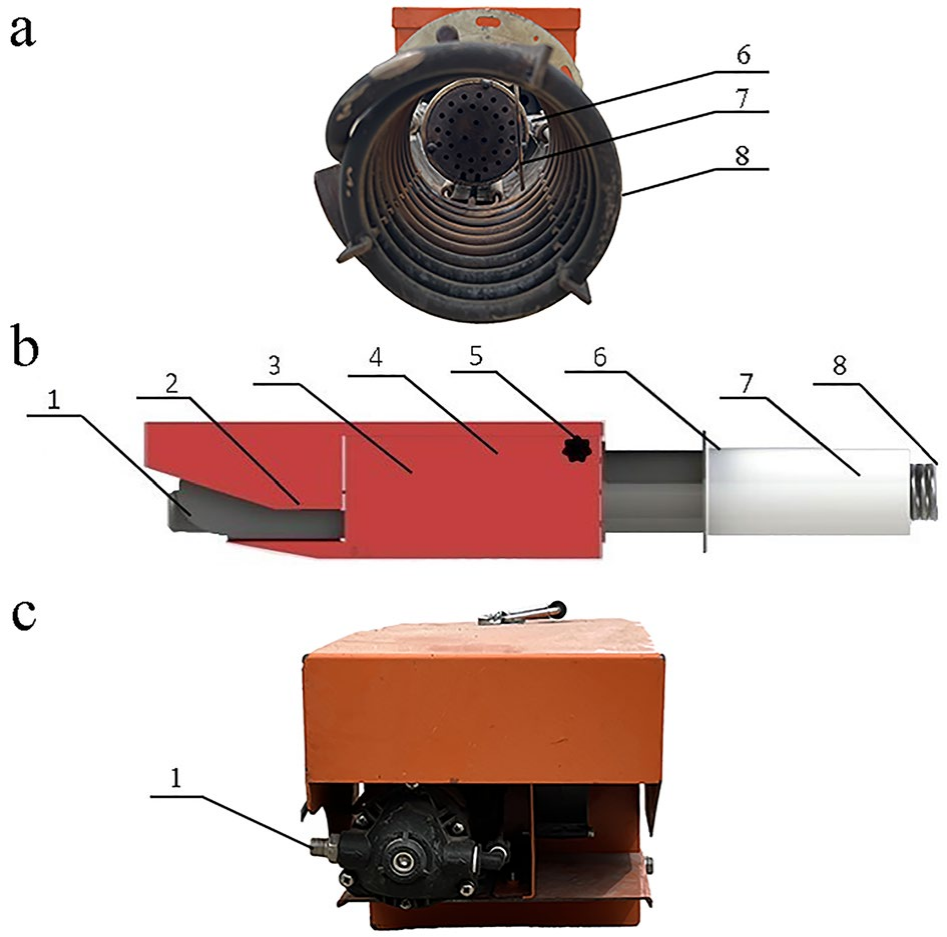
MXNY-100-1 type combustor. (**a**) Front view. (**b**) Main view. (**c**) Rear view (**d**). 1. Metal pipe connecting external alcohol-based liquid fuel storage tank, 2. Intelligent control device, 3. Heating gasification device, 4. Oil pump for alcohol-based fuel, 5. Preheat drain valve, 6. Fuel filter, 7. Combustor nozzle, 8. Interface between the combustor and the furnace.

**Figure 3:**
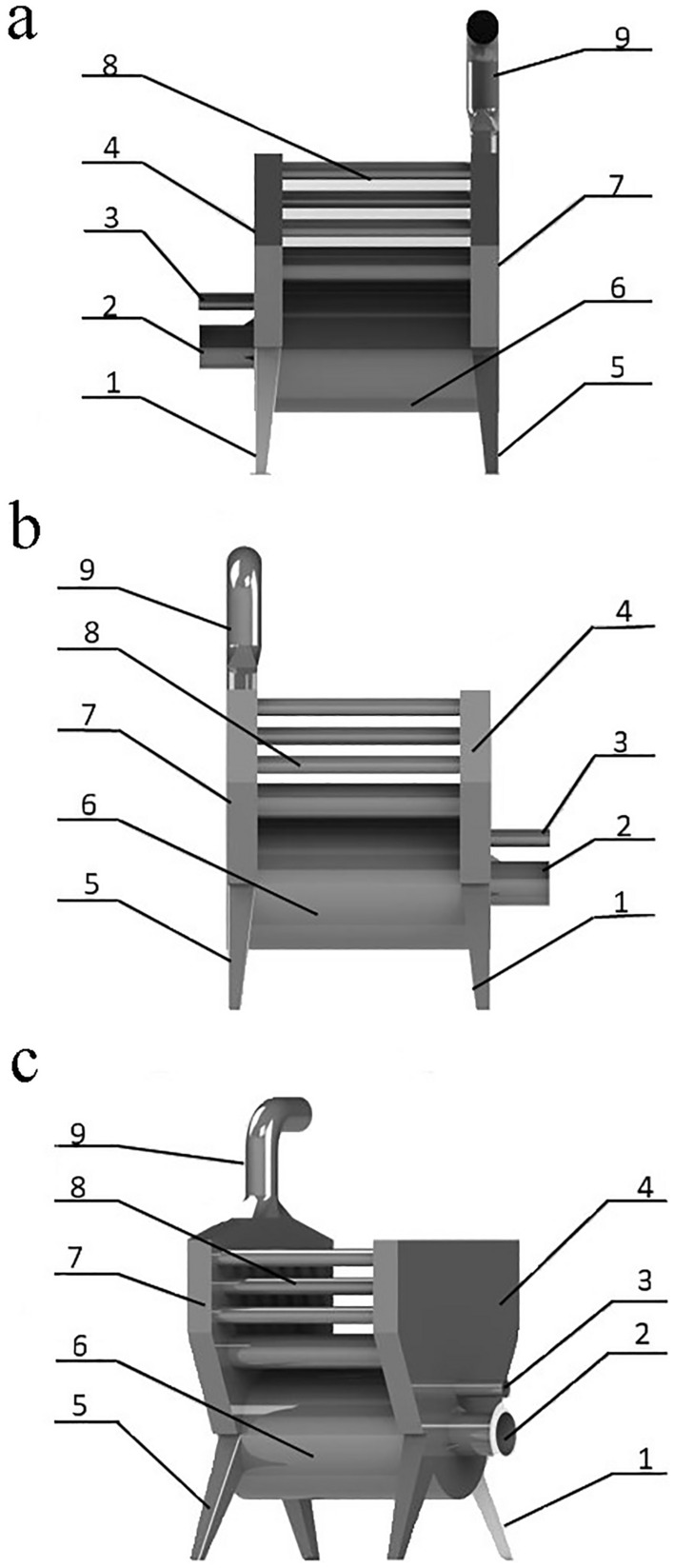
Heating dissipation zone. (**a**) Main view. (**b**) Rear view. (**c**) Left view. 1. Right bracket, 2. Interface between the furnace and the combustor, 3. Air inlet for blower, 4. Right fire box, 5. Left bracket, 6. Furnace body, 7. Left fire box, 8. Heat dissipating aluminum fin, 9. Interface for chimney.

**Figure 4:**
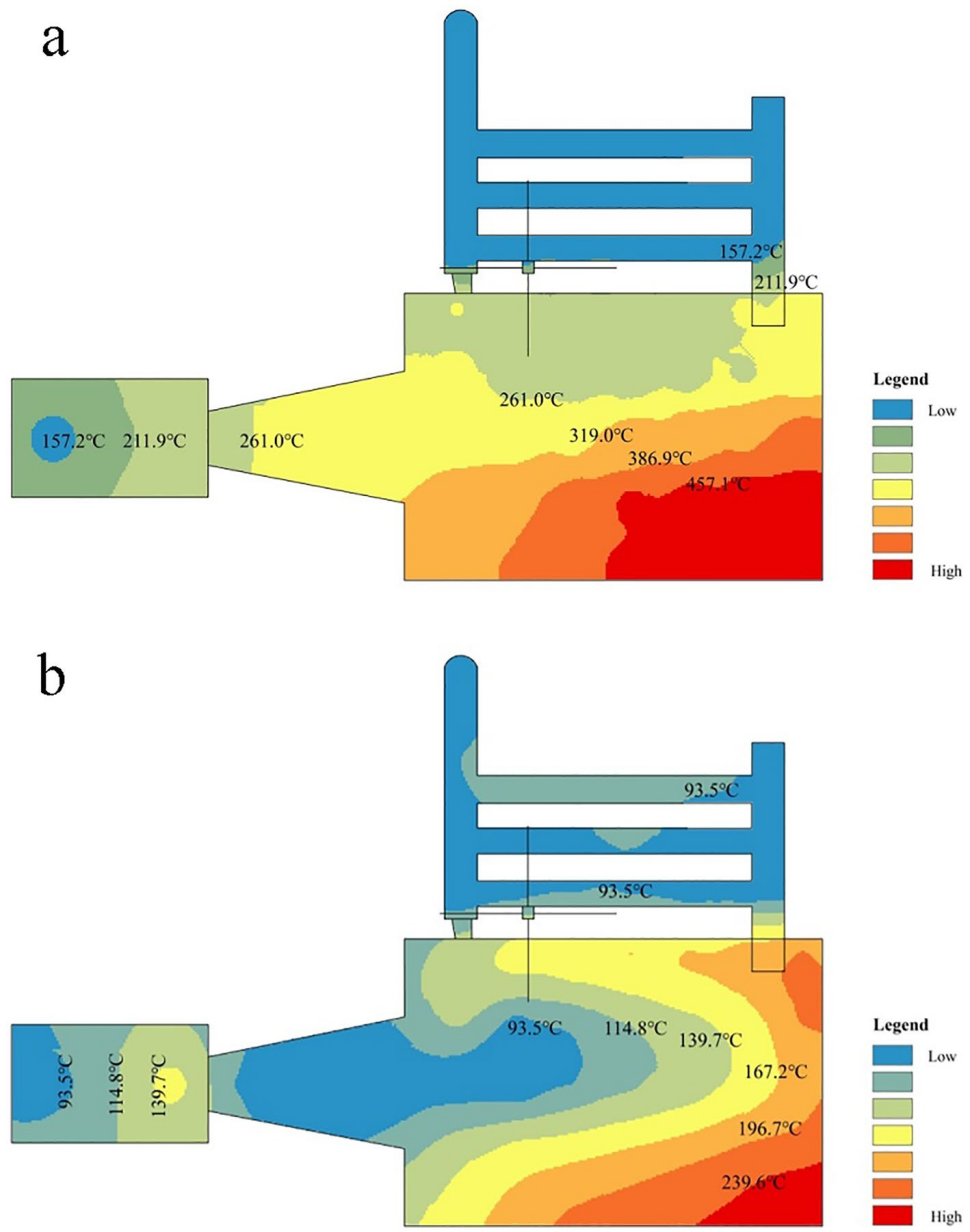
Spatial distribution of section temperature for (**a**) maximum and (**b**) minimum heating of alcohol-based energy heating equipment.

**Figure 5:**
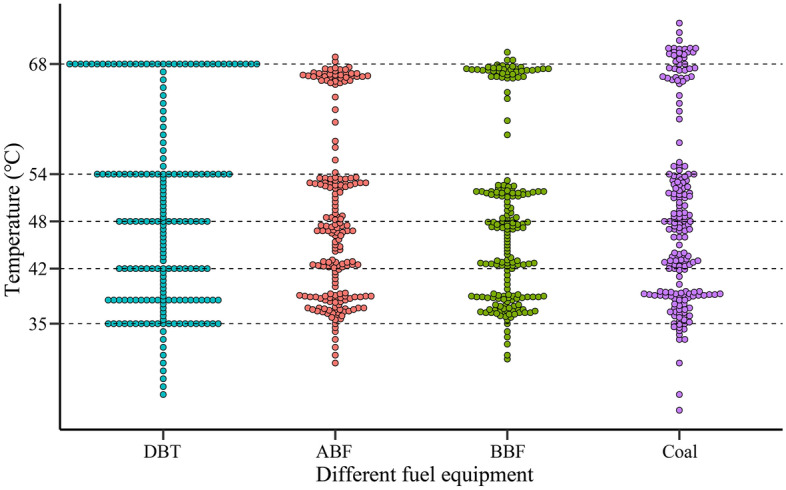
Variation curve of measured temperature in different heating equipment during tobacco curing.

**Figure 6:**
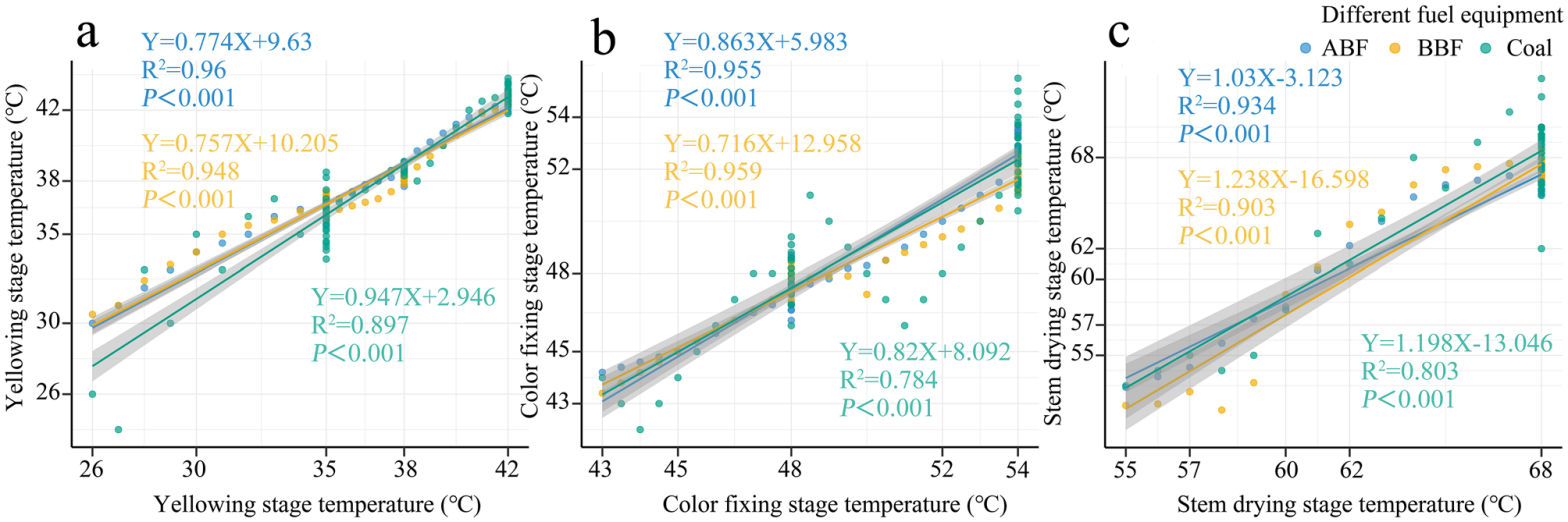
Linear regression fitting of measured temperature in different heating conditions. (**a**) Yellowing stage temperature of tobacco curing. (**b**) Color fixing stage temperature of tobacco curing. (**c**) Stem drying stage temperature of tobacco curing.

**Figure 7:**
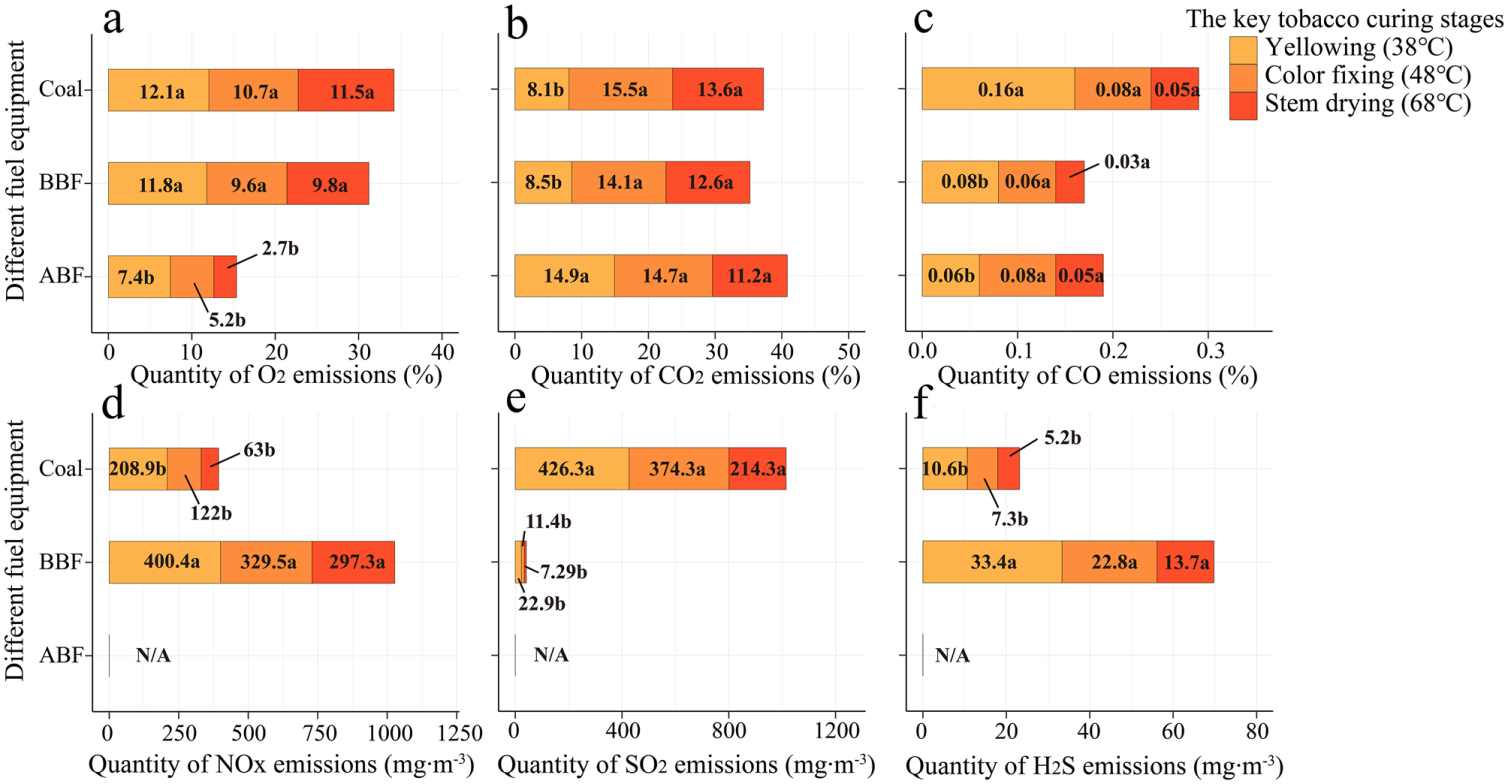
Quantity of gas emissions from chimney exit at the key TC stages. Means with different letters are statistically different from each other at p ≤ *0.05.* (**a**) Quantity of O_2_ emissions by different fuel equipment. (**b**) Quantity of CO_2_ emissions by different fuel equipment. (**c**) Quantity of CO emissions by different fuel equipment. (**d**) Quantity of NO_X_ emissions by different fuel equipment. (**e**) Quantity of SO_2_ emissions by different fuel equipment. (**f**) Quantity of H_2_S emissions by different fuel equipment.

**Figure 8:**
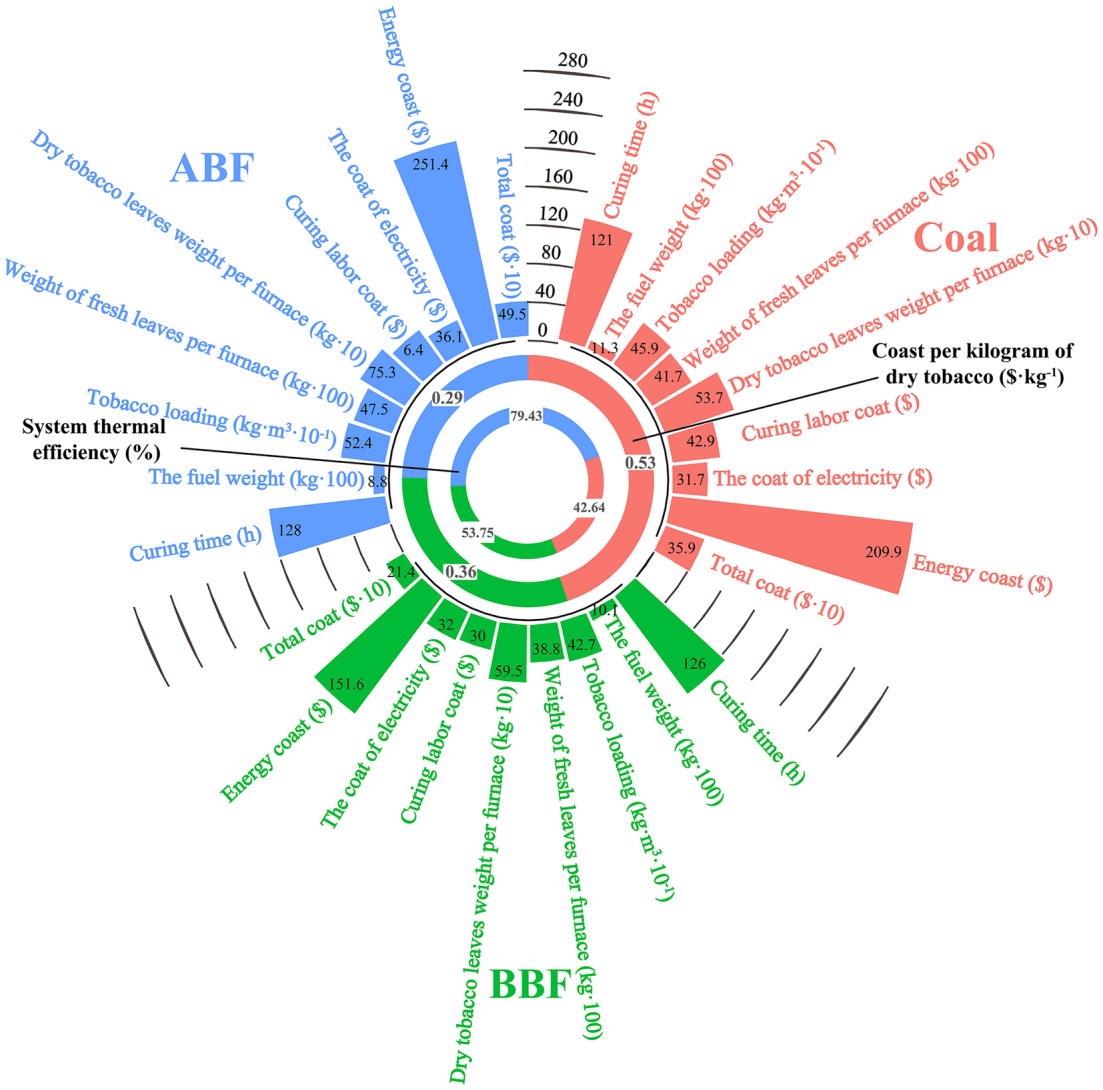
Cost of tobacco curing and system thermal efficiency. According to local market prices in 2019, coal was $185.71 (t), biomass briquettes fuel was $150 (t), alcohol-based fuel was 285.71 (t) and electricity was $0.07 (kWh).

According to the combustion characteristics of ABF, the ABF combustion equipment is designed, which is mainly composed of four main components: MXNY-100–1 combustor, ABF gas combustion zone, heat dissipation zone and intelligent controller of curing room (Fig. 2). The main parameters of MXNY-100–1 combustion chamber are shown in Table 1. K-type thermocouples were used for temperature control. The temperature measurement position is 70 mm at the upper end of the interface between the combustor and the furnace (Figs .2, 3, 4, 5, 6, 7 and 8). Automatic temperature control equipment is used to control the combustion speed of ABF and to realize accurate temperature control.

**Table 1:** Main parameters of the MXNY-100–1 combustor.

Item	Parameter	Index
Burner	Fuel combustion value (KG/h)	≥ 12
	Calorific value of fuel combustion (MJ/kg)	≥ 19
	Rated thermal power (KW)	≥ 60
	Combustion efficiency (%)	95
	Time required for ignition completion (min)	≤ 1
	Electronic igniter output voltage (KV)	≥ 15
	Combustion fan power (W)	48
	Flame detection	Ionic formula
	Fuel pump (W)	≥ 24
	Number of nozzle holes	32
	Nozzle hole diameter (mm)	82
Combustor operating conditions	Ambient temperature (°C)	0–45
	Ambient humidity (%)	≤ 85
	Altitude (m)	≤ 3000
	Operating voltage (V)	220 ± 20% or 380 ± 20%

The ABF is pre-stored in a metal storage barrel, the external ABF storage tank is connected to the MXNY-100-1 combustor through a metal pipe (Fig. 2), the front end of the combustor is equipped with a fuel filter; the ABF is gasified and burned through a MXNY-100-1 type combustor; the MXNY-100-1 type combustor is pre-stamped by a preheated discharge valve and then ignited by the igniter to preheat the ABF, heating the gasification device, preheating the temperature and time to reach the set value, the working valve and the oil pump are opened and continuously gasified and burned. At the same time, flame monitoring during preheating or burner operation. The controller is always working, if the flame is not detected, it will automatically signal, and the controller will alarm and stop to protect.

At the same time, an independent combustion zone and heat dissipation zone (Fig. 3) are designed to provide a safe and independent combustion chamber for the combustion of the MXNY-100–1 combustor, and the heat generated by the gasification combustion of the ABF is transported to the heat dissipation zone for heat exchange. Since the combustion chamber is also in the heating chamber of the oven, the heat dissipation fins are welded on the furnace wall to increase the heat dissipation area and improve the heat transfer efficiency. Under the action of the circulating fan in the curing room, heat exchange occurs with the air in the curing room, and the heat energy produced by the gasification combustion of the volatile alcohol-based fuel provides the use of tobacco leaf curing. In order to delay the high temperature of fuel combustion in the furnace and other time into the atmosphere, steel as far as possible, the heat in the high temperature gas is washed through the circulating fan and lost to the heating room, and then recycled into the smoke loading room for tobacco leaf curing. The main component parameters of ABF equipment are shown in Table 2.

**Table 2:** Summary of main components of alcohol-based fuel equipment.

Material	Components	Spare parts (mm)	Outline size (mm)	Thickness (mm)
Furnace chamber	Head cover	φ450	4	16Mn steel
	Furnace wall	φ450*1020	2.75	16Mn steel
	Furnace cooling fin	500*40	2.75	Q235 steel
	Supporting foot	540*60*40	2.75	Q235 steel
	Explosion-proof opening	φ80*280	2.75	Q235 steel
	Combustion opening	φ220*280	2.75	Q235 steel
Heat exchanger	Front fire box	1030*520*140	2.75	Q235 steel
	Rear fire box	1030*420*140	2.75	Q235 steel
	Finned base tube (heat fin)	φ87*710	3	16Mn steel
	Fin		1	Q235 steel

### Test design

The experiment was carried out in Hongda Science Park, Dali Prefecture, Yunnan Province, from July to August 2019. Using the National Tobacco Monopoly Act No.418 of 2009, Barn A using traditional coal heating equipment; Barn B using biomass briquettes fuel integrated heating equipment; Barn C equipped with alcohol-based fuel heating equipment (Fig. 2a,b). All three flue-cured tobacco rooms have 100 mm thick cotton insulation embedded in the walls, it’s just the heating equipment, other facilities and equipment remain unchanged. Select the standardized cultivation of contiguous tobacco fields, and after the uniform quality of the middle leaves are mature, they will be harvested, rod-made, loading and fire-cured in the same time. In this study, the size of all dense curing barns is length × width × height = 8 m × 2.7 m × 4.2 m.

## Test method

### External surface temperature detection of heating equipment

According to the heat conduction characteristics, the temperature distribution in the furnace should be continuous during fuel combustion. In order to understand the temperature distribution of ABF equipment, the representative sides (such as front, side or back) of the equipment were measured. Therefore, under the natural condition of no circulating machine outside the curing room and the stable combustion of fuel, the data of the fuel equipment of the ABF curing room is gridded, and the distance of the point distribution is 20 × 20 cm. The data are collected by gridding the fuel equipment of the ABF curing room Using Autocad 2013 to locate the contour of heating equipment and the X–Y coordinates of sampling point space, the temperature of the sampling point is measured by infrared thermometer (Lei Qin RAYTEK-CI1B). Arcgis10.0 software is used to interpolate the distribution map of the spatial variation of the maximum and minimum heating temperature of the equipment.

### The chimney exhausts gas detection

At the key DBT of 38, 48, and 68 °C during test 1, a flue gas analyzer (RBR Ecom-J2KN, Germany) was employed to detect the oxygen (O_2_), as well as the carbon monoxide (CO), carbon dioxide (CO_2_), nitric oxide (NO), nitrogen dioxide (NO_2_), sulfur dioxide (SO_2_) and hydrogen sulfide (H_2_S) concentrations at the chimney exit, while the fuel was burning steadily during tobacco curing process.

### Curing heat efficiency and curing energy consumption

Fuel and electricity consumption were recorded during the experiments. The quality of the green and dried tobacco for each batch and barn was analyzed statistically by taking representative samples. The operating costs for the tobacco curing were equal to the local labor cost per unit time multiplied by the number of people required to fuel the furnace and remove the ash. The system thermal efficiency was calculated using Eq. (5).

### Data statistics

The 3D-Max 2013 software was used to draw the stereoscopic structure effect diagram of the equipment. Statistical analysis were performed by two-way analyses of variation (ANOVAs) using SPSS22.0 (SPSS Institute Inc.). The 3D-Max 2013 software was used for drafting, and the GraphPad Prism 5.0 software was used for experimental data analysis. Linear regressions of the best-fitting models (*P* < 0.05) according to the R platform (version 4.0.3) analysis were conducted to investigate the relationship between DBT and different fuel equipment (ABF, BBF, Coal) of barn coal during yellowing, color fixing and drying stages during the process of curing temperature stabilization.

## Results and analysis

### Temperature distribution of barn ABF during heating

In the heating outdoor of the bulk curing barn and under the condition of no wind, when the alcohol-based energy equipment is in stable combustion of fuel, the grid detection results of the side temperature of the equipment at maximum and minimum heating are shown in Fig. 4. The heating equipment profile presents a continuous temperature hierarchical distribution, from the tail of the spindle structure to the outlet of the connecting chimney, showing a trend of first increasing and then decreasing. There is a region with the highest temperature, which occurs in the gasification gas combustion region. The highest temperature at the maximum heating is about 460 °C (Fig. 4a), and the highest temperature at the minimum heating is about 240 °C (Fig. 4b). The heat dissipation load of a group of 3 radiator pipes connected to the gas combustor is heavy and located in the sub-high temperature area (250 °C), so the material selection and welding process of their parts should be strictly regulated. Under normal circumstances, the temperature of TC continues to rise in the continuous interval during the setting period of room temperature (68 °C), which can meet the requirements of curing. In order to reduce the weight of equipment and heat dissipation, the use of refractory lining material can be reduced or reduced by adding dye to the furnace door. In the absence of the use of a circulation blower forced heat dissipation, only by natural heat dissipation, the temperature at the end of the heating equipment butting the chimney is below 150 °C, indicating that the ABF heating equipment (stainless steel) designed in this research has a better heat dissipation effect.

### Analysis of temperature control capacity of heating equipment

In the process of loading and curing in bulk curing barn, Fig. 5 shows the temperature rising and stabilizing of middle tobacco leaves in three kinds of curing barn. Most of the actual temperatures in barn ABF, barn BBF and barn coal are different from those set by curing technicians at the selected time points, which indicates that the three kinds of curing barn cannot strictly follow the ideal temperature and humidity requirements, among which, the temperature difference from the set yellowing stage is ± 3.0 °C, the difference in color fixing period is ± 2.0 °C, and the maximum difference in dry gluten stage is ± 4.0 °C. In the whole curing process, the continuous curve of three kinds of curing barn around the set process curve presents the curve peak and trough swing. The deviation of temperature curve fluctuation between alcohol-based barn and biomass curing barn is less than that of coal-fired barn, which is close to the curing curve designed by technicians. It shows that the temperature rises and temperature stabilizing operation control of barn ABF and barn BBF can be controlled according to the target DBT curve preset by technicians, and the accuracy of temperature control is better than that of barn coal. It can be seen from Fig. 5 that the technical personnel can better control the equipment temperature of barn ABF and barn BBF when operating the three kinds of curing barn to heat tobacco leaves, which is more conducive to TC according to the target DBT curve.

Based on DBT curing process, R platform was used to conduct linear regression fitting on the measured temperatures of barn ABF and barn BBF, barn ABF and barn coal during yellowing, color fixing and drying stages during the process of curing temperature stabilization (Fig. 6). The determination coefficient of fitting model for barn ABF and barn BBF at any stable temperature period was r^2^ > 0.9, while the coefficient of fitting model of coal was less than 0.9. It is clear that the temperature control accuracy of barn ABF and barn BBF is generally similar, while the temperature control ability of barn coal is far away from that of barn ABF, and the performance of heat supply and temperature control is relatively poor, which may be related to the intermittent addition of coal, which leads to large temperature fluctuation.

According to the fitting curves of different curing stages, the accuracy of temperature control in the whole curing stage was ABF > BBF > Coal, and the fitting deviation of ABF in each curing stage is small and stable. In the yellowing stage (Fig. 6a), BBF and Coal showed a trend of first increasing and then decreasing fitting deviations with the increase of temperature, among which Coal showed the highest fluctuation. In the color fixing stage (Fig. 6b), BBF showed a trend of first decreasing and then increasing fitting deviation with the increase of temperature, while Coal always had a large fluctuation. In the stem drying stage (Fig. 6c), with the increase of temperature, the fitting deviations of BBF and Coal showed a trend of first increasing, then decreasing and then increasing, among which Coal showed the highest fluctuation.

### Comparative analysis of chimney exhaust emission

It can be seen from Fig. 7 that during the whole process of dense flue curing, the emission of environmental pollutants (NO_X_, SO_2_ and H_2_S) from flue gas of different energy intensive flue curing chimneys presents a continuous downward trend, with the maximum emission concentration in the yellowing stage and the minimum emission concentration in the drying period. Among them, quantity of NO_X_ emission is 10.6–400.4 mg·m^−3^, quantity of SO_2_ emission is 7.3*–*426.3 mg·m^−3^, quantity of H_2_S emission is 5.2*–*33.4 mg·m^−3^. There is a great difference in the emission of environmental pollution gases (NOx, SO_2_ and H_2_S) from different energy sources in the process of intensive curing. Among them, no NOx, SO_2_ and H_2_S are detected in the tail gas of barn ABF, while the SO_2_ concentration in the tail gas of barn coal exceeds 400 mg·m^−3^ in the yellowing period (38 °C), and the NOx concentration in the tail gas of barn BBF exceeds 400 mg·m^−3^ in the yellowing period (38 °C). According to the integrated emission standard of air pollutants of the people's Republic of China (GB16297-1996), the SO_2_ emission concentration is significantly lower than the maximum allowable emission concentration (1200 mg·m^−3^), but the NOx concentration is second only to the maximum allowable emission concentration (420 mg·m^−3^), indicating that the barn ABF is more green and environmental friendly than other energy intensive curing.

In the process of dense flue curing, the heat required in different stages of flue curing is different, and the O_2_ concentration required by different energy sources in the combustion process is also different. The contents of gas components (O_2_, CO_2_ and CO) at the key curing temperature points are shown in Fig. 7. There are significant differences in O_2_ concentration at flue curing yellowing stage, color fixing stage and drying gluten stage among different energy sources (*P* < 0.05), while CO_2_ and CO concentration only have significant difference in curing yellowing stage (*P* < 0.05). In terms of O_2_ concentration at chimney outlet, ABF was significantly lower than BBF by 37.12% (*P* < 0.05) and Coal by 38.42% (*P* < 0.05). In terms of CO_2_ concentration at chimney outlet, ABF was significantly higher than BBF by 75.12% and Coal by 84.2% during the yellowing period (*P* < 0.05). In terms of CO concentration at chimney outlet, ABF was 25% lower than BBF and 62.5% significantly lower than Coal in yellowing phase (*P* < 0.05). The results show that the oxygen consumption of ABF is higher than that of BBF and coal during curing, which proves that the liquid fuel is fully burned. The CO concentration at the chimney outlet of barn coal is between 0.05 and 0.16%, which is far higher than that of other energy curing barn, which is not conducive to the full utilization of fuel and serious energy waste.

### Comparison and analysis of heating equipment Curing energy consumption and system thermal efficiency

In the process of loading and curing in bulk curing barn, it can be seen from Fig. 8 that the cost per kilogram of tobacco in each curing barn of barn ABF is lower than that of barn coal and barn BBF, and the average value is $0.24 and $0.07 lower than that of barn coal and barn BBF, respectively. In the process of curing fuel labor operation, the barn ABF uses automatic equipment to accurately control the quantitative combustion of liquid fuel, which significantly reduces the labor cost of manual curing compared with barn coal and barn BBF. Compared with the barn BBF and barn coal, the coast per kilogram of dry tobacco was reduced by 19.44% and 45.28%, respectively. In the process of TC, the high-temperature exhaust gas above 100 °C at the outlet of the chimney is discharged through the chimney, resulting in the loss of curing heat, which is lower than nearly 80% of the thermal efficiency of the barn ABF. Compared with the barn BBF and barn coal, the thermal efficiency of barn ABF heating equipment in the barn was increased by 44.78% and 86.28%, respectively. Additionally, the coast per kilogram of dry tobacco was reduced by 19.44% and 45.28%, respectively.

## Discussion

TC requires high temperature accuracy, different flue-cured tobacco varieties and different curing stages require different temperatures. If the temperature is not controlled properly, the quality of flue-cured tobacco will be seriously affected. Therefore, researchers aim at combining different fuel types to study the intelligent TC based on different qualities of fresh tobacco leaves and the temperature and humidity control characteristics of different curing stages^5,29^. In this study, compared with the barn coal, the barn ABF and barn BBF can control the heating and stabilizing operation more accurately according to the target DBT curve preset by the technicians. Therefore, it may be possible to achieve intelligent tobacco curing and intelligent drying of different agricultural and sideline products (fruits, vegetables, and grains) via the use of barn ABF and barn BBF. The TC is a labor-consuming and time-consuming link and requires constant refueling to maintain the temperature within an appropriate range without interruption. During the curing process of barn ABF, the ABF can be added at one time according to the temperature requirements of the target DBT curve, which can effectively eliminate the labor operation of adding fuel, realize unattended operation, and achieve the purpose of accurately controlling the indoor temperature of the barn. Wang and Duan in 2017^14^ found that the system thermal efficiency of barn ABF was significantly higher than that of barn coal by integrating “Internet + ” remote monitoring and management platforms; and realized real-time monitoring of fuel usage and equipment operation status and other data information; automatically adjust the size of firepower, precisely control the curing temperature, and improve the heat utilization efficiency of ABF; after a failure, there are safeguard measures to judge and display the range of failures, thus improving maintenance efficiency of equipment. Our study shows that intelligent curing can be achieved by modifying barn ABF.

The combustion equation of ABF is C_x_H_y_O_w_ + (x + y/4-w/2)O_2_ → x CO_2_ + y/2H_2_O + Q calories (molecular formula is 2CH_3_OH + 3O_2_ = 2CO_2_ + 4H_2_O), which shows that the emissions from the combustion of ABF are CO_2_ and H_2_O, and it will not produce SO_2_, CO, NO_X_ and other harmful gases and smoke^30^. Some studies showed that CO_2_ from the combustion of BBF just provides the amount of CO_2_ needed for biomass growth^31^. In this study, the CO_2_ from barn ABF during the yellowing period was significantly higher than that of barn BBF and barn coal, while CO was significantly lower than that of barn coal, indicating that the combustion of ABF was relatively complete. Due to the extremely low sulfur content of ABF, it is difficult to detect the quantity of SO_2_ and H_2_S, which further confirming that ABF is a potential clean fuel. The reasons why the barn ABF did not produce NO_X_ may include that the ABF does not contain nitrogen oxides, the ABF and air are less mixed in the early stage, and the peak temperature of ABF combustion is lower^32,33^. In this study, the BBF burning is also produced a lot of pollutant gas. However, some studies have shown that BBF combustion produces less pollutant gas^34^, which may be due to the emissions from the combustion of BBF are related to biomass raw materials. To reduce pollutant gas emissions, desulfurization and denitrification treatment to BBF is required. In addition, the ABF with the particulate matter (PM) emission reducing ability can be used as engine fuel additives; it can also be used as automobile engine fuels instead of gasoline to greatly reduce the atmosphere caused by automobile travel Pollution^35,36^. The ABF has high anti-knock performance and high ignition point, and is not prone to fire accidents. Its flash point is 20 °C and can be destroyed with water, which is safer than using natural gas fuel and coal gas fuel.

The thermal efficiency of the barn ABF is significantly higher than that of barn BBF and barn coal. It may be the self-oxygen effect of the ABF, which promote the complete combustion of ABF; besides, it may also be the barn ABF has a better radiator, thereby improving the overall thermal efficiency, and the energy released by the combustion of ABF can be fully utilized. CO is an intermediate product produced by insufficient combustion of hydrocarbons^37,38^. The comparison of outlet gas temperature and CO content shows that the combustion efficiency of barn ABF is higher than that of barn BBF and barn coal. Liu in 2020^31^ used different energy sources to study energy research on grains drying, and found that the heat conversion rate of barn ABF hot blast stoves reached more than 90%, which has the characteristics of high efficiency, energy saving, and environmental protection. Studies have shown that ABF has a higher octane value and a higher anti-knock performance, which is an excellent additive to improve the octane value of gasoline^19,39,40^. However, ABF also has the characteristics of low calorific value, which is only 60% of some high-calorific value fuels, it is sufficient for TC, but for other curing that requires higher calorific value, it is necessary to increase the calorific value of ABF. There have been some researches on increasing the calorific value of ABF. For example, adding stabilizing agent can improve the stability of fuel combustion and greatly increase the calorific value; adding a catalyst ferrocene (de-iron) can increase the combustibility of the fuel, achieve full combustion, increase the calorific value, and avoid air pollution; Adding smoke suppressor, a corrosion inhibitor, and a smell removing agent can avoid the generation of fuel residual liquid, avoid the generation of smoke, odor and coke, and have a long storage time^10^. In addition, Csemany et al.^41^, determined thermophysical and transport properties of different volume fractions n-butanol, mixture of acetone-butanol-ethanol biofuels and their blends with standard diesel oil. This study considered that the combustion characteristics of 25% volume fraction of biofuel for n-butanol and mixture of acetone-butanol-ethanol biofuels blends are advantageous. This study provides a new way to reduce the cost of alcohol-based fuel, improve combustion efficiency and the emission of CO and unburnt hydrocarbon emission.

## Conclusion

Under the conditions of this study, the barn ABF can meet the highest temperature requirements of TC. Compared with barn BBF and barn coal, it is more conducive to increase temperature and stable temperature according to target DBT curve during TC curing process; Meanwhile, CO_2_ and CO were produced during the combustion of ABF, but no harmful gases such as NO_X_, SO_2_ and H_2_S are detected in the exhaust gas of the chimney; The thermal efficiency of the barn ABF in the TC process reaches 79.43%, and the cost per kilogram of tobacco leaves is lower than that of barn BBF and barn coal. Therefore, compared to barn coal and barn BBF, the barn ABF can more accurately control temperature changes, and has obvious advantages in environmental protection and heat utilization efficiency.

## Data Availability

All data, models, and code generated or used during the study appear in the submitted article.
